# Analysis of care-seeking and diagnosis delay among pulmonary tuberculosis patients in Beijing, China

**DOI:** 10.3389/fpubh.2024.1369541

**Published:** 2024-04-16

**Authors:** Lijie Zhang, Xiaoge Ma, Hanqing Gao, Cheng Bao, Yue Wu, Sihui Wu, Menghan Liu, Yuhong Liu, Liang Li

**Affiliations:** ^1^Beijing Chest Hospital, Capital Medical University, Beijing, China; ^2^Beijing Tuberculosis and Thoracic Tumor Research Institute, Clinical Center on Tuberculosis, China CDC, Beijing, China; ^3^Department of Epidemiology, School of Public Health, Cheeloo College of Medicine, Shandong University, Jinan, China; ^4^Institute of Tuberculosis Prevention and Control, Tongzhou District Center for Disease Prevention and Control, Beijing, China; ^5^Beijing Changping Institute for Tuberculosis Prevention and Treatment, Beijing, China

**Keywords:** pulmonary tuberculosis, delay, diagnosis, influencing factors, logistic regression

## Abstract

**Background:**

Tuberculosis (TB) remains a significant public health challenge in China. Early detection and diagnosis of TB cases are crucial to interrupt disease transmission and prevent its progression. This study aims to describe the delay in seeking care and diagnosis among patients with pulmonary tuberculosis (PTB) and identify the influencing factors in two counties in Beijing.

**Methods:**

A retrospective analysis was carried out to investigate care-seeking and diagnosis delay in two counties in Beijing. Basic information of PTB patients from January 1 to December 31, 2021, was extracted from the Tuberculosis Information Management System of China (TBIMS), and all enrolled patients were interviewed via telephone using a standard questionnaire. Statistical description was performed using the median and interquartile range (IQR). Chi-square test and multivariate logistic regression model were used to analyze the influencing factors.

**Results:**

537 patients were enrolled. The median duration of care-seeking and diagnosis delay was 11 (IQR: 5–26) days and 8 (IQR: 0–18) days, with 41.71 and 35.20% of patients experiencing delays (>14 days). The study found that being asymptomatic (OR = 2.791, 95%CI: 1.710–4.555) before seeking medical care and not attending work during treatment (OR = 2.990, 95%CI: 1.419–6.298) were identified as risk factors for care-seeking delay. Patients who were tracked (OR = 2.632, 95%CI: 1.062–6.521) and diagnosed at tuberculosis control and prevention institutions (OR = 1.843, 95%CI: 1.061–3.202) had higher odds of diagnostic delays. 44.69% of patients presented a total delay (>28 days), with a median duration of 25 (IQR: 13–39) days. A multivariate logistic regression analysis showed that healthy examination (OR = 0.136, 95%CI: 0.043–0.425) was a protective factor for total delay.

**Conclusion:**

Public interventions are necessary to improve the efficiency of PTB patients detection and treatment in Beijing. Medical services should focus on the target population and improve access to medical care to further reduce delays for PTB patients.

## 1 Introduction

Tuberculosis (TB) is an airborne infectious disease caused by *Mycobacterium tuberculosis* (*M.tb.*) (1, 2). It is concerning that TB has reemerged as the primary cause of death among infectious diseases globally, following the decline in mortality rates due to COVID-19 (3). According to global statistics, around a quarter of the world’s population is infected with *M.tb.* (4). The 2023 Tuberculosis Report by the World Health Organization (WHO) states that the COVID-19 pandemic resulted in 7.5 million new tuberculosis cases globally in 2022, the highest number since the WHO began global TB epidemic surveillance in 1995. China is among the countries with a high burden of TB and drug-resistant TB. Among the 30 countries with a significant TB burden, China ranks third globally in estimated TB cases, accounting for 7.1% of the total TB cases worldwide, following India (27%) and Indonesia (10%) (5).

During the period from symptom onset to treatment initiation, patients with pulmonary tuberculosis (PTB) pose a substantial risk for transmission due to their high infectivity (6, 7). However, inadequate awareness among PTB patients significantly contributes to delays in seeking medical care and diagnosis, thereby greatly amplifying the risk of *M.tb.* transmission within populations (8). The Fifth TB Epidemiological Sample Survey in China found that over 50% of patients with symptomatic PTB did not promptly seek medical care (9).

Delay in seeking medical help, diagnosis and treatment by healthcare institutions are prevalent among PTB patients in both high and low TB burden regions globally (10–12). In low- and middle-income countries, up to 42% of patients exhibit delayed healthcare-seeking behavior upon experiencing TB-related symptoms (13), with diagnosis delay ranging from 47 to 63% (14–16). Untreated sputum positive patients can infect an average of 10 contacts per year and over 20 contacts throughout the natural course of their disease until death (17). The low detection rate in China suggests that either the healthcare system’s ability to diagnose PTB early falls short of expectations or symptomatic individuals are not actively seeking care.

Delays in seeking care from healthcare facilities and the health system’s inefficiency in identifying TB patients are the main reasons for the high burden of tuberculosis in China (18). This situation also places unnecessary economic burdens on patients’ families. In 2019, around 20% of tuberculosis patients worldwide experienced catastrophic health expenditures, which means that the costs associated with tuberculosis diagnosis and treatment exceeded 20% of the household’s annual income, both directly and indirectly (19). The tuberculosis epidemic is a significant public health issue and a complex socio-economic challenge that has significantly hindered the rapid economic and social progress of developing nations. Therefore, it is crucial to comprehend the characteristics linked to delayed diagnosis among patients with PTB and analyze the factors that affect this delay.

The objective of this study was to investigate the factors of care-seeking delay, diagnostic delay, and total delay among PTB patients in two counties of Beijing in 2021, and to propose corresponding measures for the early detection and effective management of PTB patients.

## 2 Methods

### 2.1 Study patients

A retrospective observational cross-sectional analysis was conducted on care-seeking, diagnosis, and total delay of PTB patients, based on existing study. Tuberculosis Information Management System of China (TBIMS) was used to collect all notified PTB cases in Tongzhou and Changping Counties from January 1 to December 31, 2021. Patients with extrapulmonary tuberculosis, impaired hearing, language expression and comprehension, and incorrect registration information were excluded from this study.

### 2.2 Data collection

**Figure 1:**
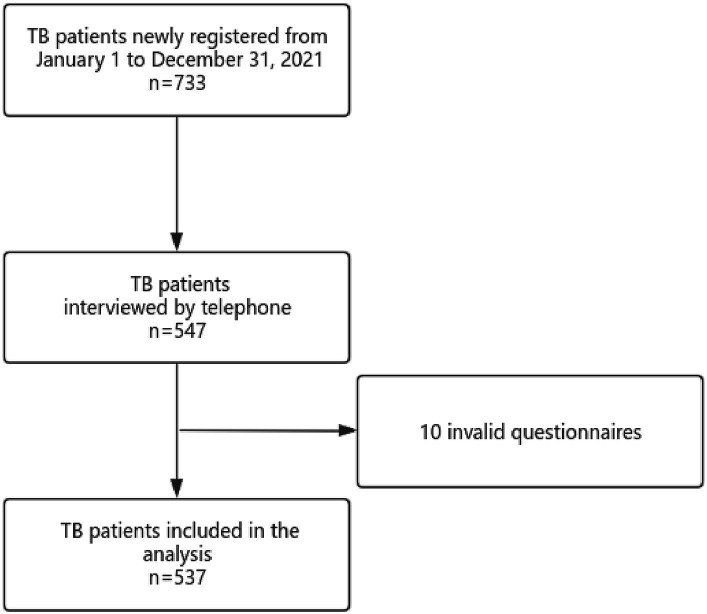
Flow chart of patient selection.

Patient demographics, including gender, age (age groups: ≤29, 30–49, 50–69, ≥70 years), occupation, high-risk population, household registration, patient source, sputum examination results, comorbidity, time of symptoms onset, time of visit, and time of diagnosis, were collected based on TBIMS. Symptoms prior to treatment, first contact unit, unit of diagnosis, number of referral hospitals, attendance at work during treatment, and number of people living together were obtained through telephonic interviews by the trained investigators using the designed standard questionnaire (Figure 1). To ensure objectivity, the investigators underwent strict and uniform training to control for bias. If patients were uncertain about their answers, we used corresponding terms to help them recall memories. If their answers were still ambiguous, they would not be included in the study. All questionnaires were coded and double-checked before entering them into the database. Epidata was used to establish the database. Each questionnaire was entered in duplicate by different entry staff, and the double database was checked and corrected to ensure the accuracy and integrity of the questionnaire data entry software.

### 2.3 Relevant definitions

According to the WHO, care-seeking delay is the time between the onset of symptoms associated with PTB and the patient’s first visit to a healthcare facility. Diagnosis delay is the duration between the patient’s first care-seeking encounter and receiving a definitive diagnosis of PTB. The total duration of both care-seeking delay and diagnosis delay is referred to as total delay (17). Following previous studies, a delay in care-seeking and diagnosis was defined as more than 14 days, while a total delay was defined as more than 28 days (20, 21).

High-risk population: This study defines the high-risk population as individuals who are HIV/AIDS patients, diabetes patients, school and kindergarten personnel, regulatory personnel, breeding personnel, dust exposure personnel/pneumoconiosis patients, residents of nursing homes/welfare homes, close contacts of pathogenic positive tuberculosis patients, medical staff, or people in other crowded places.

Household registration: It refers to whether individual is a local resident of Beijing.

According to the “Guidelines for Tuberculosis prevention and control in China” (22), register the source of tuberculosis infection through transfer, direct visit, healthy examination, referral, and tracking. (1) Transfer: It refers to the individual who went to medical and health institutions (excluding tuberculosis prevention and control institutions) after experiencing suspicious symptoms of TB, and was diagnosed as TB or suspected TB by chest X-ray or sputum examination, and brought the transfer form filled by the doctor to the tuberculosis prevention and control institutions for treatment. (2) Direct visit: It refers to the individual who proactively seek medical treatment at tuberculosis prevention and control institutions after experiencing suspicious symptoms that suggest TB. (3) Referral: It refers to the medical staff or relevant personnel who will find suspicious symptoms of TB and encourage them to go to tuberculosis prevention and control institutions for examination. (4) Tracking: It refers to the disease prevention and control institutions encouraging TB and suspected TB patients who have been reported but have not yet visited designated TB medical institutions for diagnosis and treatment.

Diagnostic results: Laboratory confirmed PTB included sputum smear positive, *M.tb.* culture positive, and molecular biology positive PTB. Not laboratory confirmed PTB was defined as sputum smear and *M.tb.* culture negative PTB and tuberculosis without sputum examination (23).

Comorbidities: Patients with diabetes, pneumoconiosis and HIV/AIDS.

### 2.4 Statistical analysis

Descriptive epidemiological methods were used to analyze the demographic characteristics. Continuous variables were presented as median and interquartile range (IQR), while categorical variables were presented as ratios and proportions. The statistical analysis was conducted using IBM SPSS 25.0 software. The Chi-squared test and multivariate logistic regression model were used to investigate the relationship between relevant influencing factors and delay among PTB patients. A *p*-value of less than 0.05 was considered statistically significant.

### 2.5 Ethical considerations

Ethics approval was obtained from the Ethics Committee, Beijing Chest Hospital, Capital Medical University, Beijing, China (2018-30-01 dated 21 August 2018). All study participants provided informed consent.

## 3 Results

### 3.1 Study population

A total of 537 patients with PTB were enrolled in this study, of whom 344 (64.1%) were male. The mean age was 40.68 ± 17.23 years. Among them, 168 (31.3%) were workers or farmers, while 334 (62.2%) were non-local. The majority of patients, 388 (72.3%) were transferred from other healthcare facilities. A total of 264 (49.2%) patients were laboratory confirmed PTB. Complications other than TB were observed in 51 (9.5%) patients. Before their visit, 199 (37.1%) patients were asymptomatic. The majority of individuals initially sought medical care at non-specialized TB hospitals, comprising 417 (77.7%), while only 87 (16.2%) patients directly visited TB specialized hospitals. Among the diagnosed institutions, TB specialized hospitals accommodated 226 (42.1%) of all the patients involved in this study. Out of all the patients, only 37 (6.9%) received a direct diagnosis at the initial medical institution. During their care-seeking process, 116 (21.6%) continued to attend work. Additionally, 85 (15.8%) of the patients resided alone, while 203 (37.8%) cohabited with one other individual (Table 1).

**Table 1:** Characterization of the studied population and univariate analysis of care-seeking, diagnosis and total delay of pulmonary tuberculosis cases (*n* = 537).

Variable	n (%)	Care-seeking delay (>14 d)			Diagnosis delay (>14 d)			Total delay (>28 d)		
		n (%)	χ ^2^	*p*-value	n (%)	χ *^2^*	*p*-value	n (%)	χ *^2^*	*p*-value
Gender			0.672	0.412		0.234	0.628		0.458	0.498
Male	344 (64.1%)	139 (40.4%)			123 (35.8%)			150 (43.6%)		
Female	193 (35.9%)	85 (44.0%)			65 (33.7%)			90 (46.6%)		
Age group			1.930	0.587		0.518	0.915		1.327	0.723
≤ 29	182 (33.9%)	82 (45.1%)			65 (35.7%)			86 (47.3%)		
30–49	197 (36.7%)	82 (41.6%)			68 (34.5%)			87 (44.2%)		
50–69	106 (19.7%)	39 (36.8%)			35 (33.0%)			47 (44.3%)		
≥ 70	52 (9.7%)	21 (40.4%)			20 (38.5%)			20 (38.5%)		
Occupations			3.911	0.689		7.852	0.249		10.276	0.114
Cadres and staff, teachers and medical staff	70 (13.0%)	29 (41.4%)			25 (35.7%)			32 (45.7%).		
Workers and farmers	168 (31.3%)	69 (41.1%)			56 (33.3%)			68 (40.5%)		
Housework and unemployment	165 (30.7%)	75 (45.5%)			68 (41.2%)			89 (53.9%)		
Retirees	47 (8.8%)	14 (29.8%)			14 (29.8%)			17 (36.2%)		
Business services staff	60 (11.2%)	25 (41.7%)			14 (23.3%)			22 (36.7%)		
Students	21 (3.9%)	9 (42.9%)			8 (38.1%)			10 (47.6%)		
Other	6 (1.1%)	3 (50.0%)			5 (83.3%)			2 (33.3%)		
High risk population			0.146	0.703		0.032	0.859		0.045	0.832
Yes	78 (14.5%)	31 (39.7%)			28 (35.9%)			34 (43.6%)		
No	459 (85.5%)	193 (42.0%)			160 (34.9%)			206 (44.9%)		
Household registration			1.302	0.254		4.265	0.039		0.052	0.820
Local	203 (37.8%)	91 (44.8%)			60 (29.6%).			92 (45.3%)		
Non-local	334 (62.2%)	133 (39.8%)			128 (38.3%)			148 (44.3%)		
Patients source			18.925	0.001		14.292	0.006		21.689	< 0.001
Health examination	32 (6.0%)	2 (6.3%)			8 (25.0%)			4 (12.5%)		
Direct visit	43 (8.0%)	22 (51.2%)			19 (44.2%)			24 (55.8%)		
Transfer	388 (72.3%)	166 (42.8%)			125 (32.2%)			169 (43.6%)		
Referral	49 (9.1%)	22 (44.9%)			20 (40.8%)			27 (55.1%)		
Tracking	25 (4.7%)	12 (48.0%)			16 (64.0%)			16 (64.0%)		
Diagnostic results			<0.001	0.983		0.964	0.326		0.485	0.486
Laboratory confirmed PTB	264 (49.2%)	110 (41.7%)			87 (33.0%)			122 (46.2%)		
Not laboratory confirmed PTB	273 (50.8%)	114 (41.8%)			101 (37.0%)			118 (43.2%)		
Comorbidity			0.266	0.606		0.438	0.508		1.551	0.213
Yes	51 (9.5%)	23 (45.1%)			20 (39.2%)			27 (52.9%)		
No	486 (90.5%)	201 (41.4%)			168 (34.6%)			213 (43.8%)		
Pre-visit symptoms			45.281	< 0.001		4.138	0.247		12.807	0.005
Cough and expectoration for >1 week <2 weeks, with chest tightness, chest pain, low-grade fever, night sweats, fatigue, loss of appetite and weight loss	116 (21.6%)	27 (23.3%)			46 (39.7%)			37 (31.9%)		
Cough and expectoration for ≥2 weeks, hemoptysis or bloody sputum	145 (27.0%)	62 (42.8%)			45 (31.0%)			76 (52.4%)		
Other	77 (14.3%)	20 (26.0%)			22 (28.6%)			31 (40.3%)		
Asymptomatic	199 (37.1%)	115 (57.8%)			75 (37.7%)			96 (48.2%)		
First contact unit			13.664	0.008		5.386	0.250		8.355	0.079
TB specialized hospitals	87 (16.2%)	48 (55.2%)			30 (34.5%)			43 (49.4%)		
Non TB-specialized hospitals	417 (77.7%)	157 (37.6%)			144 (34.5%)			179 (42.9%)		
Primary healthcare institutions	15 (2.8%)	10 (66.7%)			6 (40.0%)			10 (66.7%)		
Private practice	4 (0.7%)	2 (50.0%)			0 (0.0%)			0 (0.0%)		
Do not remember	14 (2.6%)	7 (50.0%)			8 (57.1%)			8 (57.1%)		
Unit of diagnosis			5.146	0.161		54.418	<0.001		27.128	<0.001
TB specialized hospitals	226 (42.1%)	95 (42.0%)			41 (18.1%)			73 (32.3%)		
Tuberculosis control and prevention institute	210 (39.1%)	78 (37.1%)			108 (51.4%)			106 (50.5%)		
Non TB-specialized hospitals	90 (16.8%)	46 (51.1%)			36 (40.0%)			55 (61.1%)		
Other	11 (2.0%)	5 (45.5%)			3 (27.3%)			6 (54.5%)		
Number of referral hospitals			5.786	0.122		14.709	0.002		8.067	0.045
0	37 (6.9%)	21 (56.8%)			9 (24.3%)			16 (43.2%)		
1	257 (47.9%)	108 (42.0%)			73 (28.4%)			101 (39.3%)		
2	199 (37.1%)	74 (37.2%)			87 (43.7%)			97 (48.7%)		
3 or higher	44 (8.2%)	21 (47.7%)			19 (43.2%)			26 (59.1%)		
Attendance at work during treatment			11.638	0.009		6.949	0.074		12.903	0.005
Yes	116 (21.6%)	50 (43.1%)			34 (29.3%)			47 (40.5%)		
No	266 (49.5%)	105 (39.5%)			87 (32.7%)			112 (42.1%)		
No attendance required	100 (18.6%)	35 (35.0%)			44 (44.0%)			44 (44.0%)		
Occasionally	55 (10.2%)	34 (61.8%)			23 (41.8%)			37 (67.3%)		
Number of people living together			1.905	0.592		8.209	0.042		5.904	0.116
Living alone	85 (15.8%)	41 (48.2%)			24 (28.2%)			41 (48.2%)		
1	203 (37.8%)	83 (40.9%)			72 (35.5%)			86 (42.4%)		
2	160 (29.8%)	63 (39.4%)			68 (42.5%)			81 (50.6%)		
3 or higher	89 (16.6%)	37 (41.6%)			24 (27.0%)			32 (36.0%)		

### 3.2 Influencing factors of care-seeking delay for PTB patients

The median duration for seeking medical care was 11 (IQR: 5–26) days, with 41.71% of patients experiencing a care-seeking delay (>14 days). The results of the univariate analysis showed statistically significant differences in patient source, pre-visit symptoms, first contact unit, and whether patients attended work during treatment (*p* < 0.05).

The results of the multivariate analysis results showed that health examination (OR = 0.033, 95%CI: 0.008–0.147), experiencing cough and expectoration for a duration of more than one but less than 2 weeks prior to the visit, as well as presenting with symptoms such as chest tightness, chest pain, low fever, night sweats, fatigue, loss of appetite, and weight loss (OR = 0.378, 95%CI: 0.215–0.665) were identified as protective factors associated with delayed care-seeking behavior. Conversely, the study found that being asymptomatic (OR = 2.791, 95%CI: 1.710–4.555) and not needing to attend work during treatment (OR = 2.990, 95%CI: 1.419–6.298) were identified as risk factors for delaying care-seeking (Table 2).

**Table 2:** Multivariate logistic analysis of care-seeking delay of pulmonary tuberculosis cases (*n* = 537).

Variable	*β*	$S_{\bar{x}}$	*χ^2^*	*p*-value	*OR*	95% *CI*	
						Lower limit	Upper limit
Patients source							
Transfer					1.00		
Direct visit	0.081	0.371	0.048	0.827	1.084	0.524	2.242
Health examination	3.402	0.758	20.161	<0.001	0.033	0.008	0.147
Referral	0.001	0.341	0.000	0.998	1.001	0.513	1.951
Tracking	0.097	0.451	0.046	0.830	1.102	0.455	2.666
Pre-visit symptoms							
Cough with sputum for ≥2 weeks, hemoptysis, or bloody sputum					1.00		
Cough and expectoration for more than 1 week and less than 2 weeks, with chest tightness, chest pain, low-grade fever, night sweats, fatigue, loss of appetite and weight loss	0.972	0.287	11.431	0.001	0.378	0.215	0.665
Others	0.592	0.331	3.197	0.074	0.553	0.289	1.059
Asymptomatic	1.027	0.250	16.875	< 0.001	2.791	1.710	4.555
First Visiting unit							
TB specialized hospital					1.00		
TB non-specialized hospital	0.548	0.285	3.691	0.055	0.578	0.331	1.011
Primary healthcare institutions	0.818	0.640	1.636	0.201	2.266	0.647	7.938
Private practice	0.315	1.126	0.078	0.780	0.730	0.080	6.635
Do not remember	0.033	0.657	0.003	0.959	1.034	0.285	3.747
Attendance at work during treatment							
Yes					1.00		
No	0.066	0.260	0.065	0.799	1.069	0.641	1.780
No attendance required	1.095	0.380	8.303	0.004	2.990	1.419	6.298
Occasionally	0.010	0.321	0.001	0.975	0.990	0.528	1.856

### 3.3 Influencing factors of diagnosis delay for PTB patients

The median time for diagnosing PTB patients was 8 (IQR: 0–18) days, with 35.20% of patients experiencing a diagnosis delay (>14 days). Univariate analysis results showed statistically significant differences in the delay rate of diagnosis based on household registration, diagnosing hospital unit, number of referral hospitals, and number of cohabiting patients (*p* < 0.05).

The multivariate analysis results showed that being admitted to a tuberculosis specialized hospital (OR = 0.426, 95%CI: 0.236–0.767) acted as a protective factor against diagnosis delay. Additionally, tracking (OR = 2.632, 95%CI: 1.062–6.521) and receiving the final diagnosis from an institution within the tuberculosis control and prevention institute (OR = 1.843, 95%CI: 1.061–3.202) were identified as risk factors for delayed diagnosis (Table 3).

**Table 3:** Multivariate logistic analysis of diagnostic delay of pulmonary tuberculosis cases (*n* = 537).

Variable	*β*	$S_{\bar{x}}$	*χ^2^*	*p*-value	*OR*	95% *CI*	
						Lower limit	Upper limit
Household registration							
Non-local					1.00		
Local	0.330	0.210	2.486	0.115	0.719	0.476	1.084
Patients source							
Transfer					1.00		
Direct visit	0.142	0.350	0.165	0.685	1.152	0.581	2.287
Health examination	0.025	0.452	0.003	0.956	0.976	0.402	2.367
Referral	0.313	0.333	0.881	0.348	0.731	0.380	1.406
Tracking	0.968	0.463	4.368	0.037	2.632	1.062	6.521
Unit of diagnosis							
TB non-specialized hospital					1.00		
TB specialized hospital	0.854	0.300	8.099	0.004	0.426	0.236	0.767
Tuberculosis Control and Prevention Institute	0.611	0.282	4.702	0.030	1.843	1.061	3.202
Others	0.471	0.738	0.408	0.523	0.624	0.147	2.651
Number of referral hospitals							
0					1.00		
1	0.195	0.434	0.202	0.653	0.823	0.351	1.927
2	0.051	0.454	0.013	0.910	0.950	0.390	2.312
3 or higher	0.139	0.532	0.068	0.794	1.149	0.405	3.256
Number of cohabitants							
Living alone					1.00		
1	0.234	0.306	0.588	0.071	1.264	0.694	2.302
2	0.573	0.317	3.267	0.743	1.774	0.953	3.301
3 or higher	0.119	0.364	0.107	0.206	1.127	0.552	2.302

### 3.4 Influencing factors of total delay for PTB patients

Among 537 patients diagnosed with PTB, the median duration of total delay was 25 days (IQR: 13–39), with 44.69% of patients experiencing a total delay (>28 days). Univariate analysis showed statistically significant differences in delay rates based on patients’ pre-visit symptoms, diagnostic units, number of referral hospitals, and whether they attended work during treatment (*p* < 0.05).

Multivariate analysis showed that patients who were informed through health examinations (OR = 0.136, 95%CI: 0.043–0.425), experienced cough and expectoration for a duration of more than 1 week but less than 2 weeks prior to hospital visit, and presented with at least one symptom such as chest tightness, chest pain, low fever, night sweats, fatigue, loss of appetite or weight loss (OR = 0.370, 95%CI: 0.217–0.632) had a shorter total delay in seeking medical attention for pulmonary tuberculosis diagnosis. Furthermore, the study found that being diagnosed in a specialized tuberculosis hospital (OR = 0.287, 95%CI: 0.162–0.507) is a protective factor against total delay while not requiring attendance at work during treatment (OR = 3.008; 95%CI: 1.456–6.203) emerged as a risk factor for prolonged delay in seeking medical care (Table 4).

**Table 4:** Multivariate logistic analysis of total delay of pulmonary tuberculosis cases (*n* = 537).

Variable	*β*	$S_{\bar{x}}$	*χ^2^*	*p*-value	*OR*	95% *CI*	
						Lower limit	Upper limit
Patients source							
Transfer					1.00		
Direct visit	0.101	0.356	0.080	0.777	1.106	0.550	2.225
Health examination	1.995	0.582	11.772	0.001	0.136	0.043	0.425
Referral	0.220	0.339	0.420	0.517	1.246	0.641	2.421
Tracking	0.249	0.467	0.286	0.593	1.283	0.514	3.203
Symptoms before presentation							
Cough and expectoration for ≥2 weeks, hemoptysis or bloody sputum					1.00		
Cough and expectoration for more than 1 week and less than 2 weeks, accompanied by chest tightness, chest pain, low-grade fever, night sweats, fatigue, loss of appetite and weight loss	0.994	0.273	13.236	< 0.001	0.370	0.217	0.632
Others	0.191	0.318	0.359	0.549	0.826	0.443	1.543
Asymptomatic	0.280	0.249	1.264	0.261	1.323	0.812	2.156
Diagnostic unit							
TB non-specialized hospital					1.00		
TB specialized hospital	1.250	0.291	18.432	<0.001	0.287	0.162	0.507
Tuberculosis control and prevention institute	0.535	0.292	3.353	0.067	0.585	0.330	1.038
Others	0.175	0.682	0.066	0.797	0.839	0.220	3.197
Number of referral hospitals							
0					1.00		
1	0.251	0.389	0.417	0.519	0.778	0.363	1.667
2	0.165	0.416	0.158	0.619	0.848	0.375	1.914
3 or higher	0.206	0.505	0.166	0.683	1.229	0.457	3.304
Attendance at work during treatment							
Yes					1.00		
No	0.000	0.251	0.000	0.999	1.000	0.612	1.635
No attendance required	1.101	0.369	8.902	0.003	3.008	1.456	6.203
Occasionally	0.270	0.307	0.774	1.310	1.310	0.718	2.391

## 4 Discussion

Based on a global meta-analysis, it was found that the average duration for seeking medical attention among 14 low-income countries with high TB prevalence was 28 days, while it was only 10 days in high-income nations (18). Our study shows that the median duration of seeking care for PTB patients in Tongzhou and Changping Districts of Beijing in 2021 was 11 (IQR: 5–26) days. Compared to international studies, the duration of this data is shorter than that reported in India (25 days) (24) and Portugal (33 days) (25). The median delay in diagnosing PTB patients was 8 (IQR: 0–18) days, which is shorter than the average of 14 (IQR: 4–50) days observed in Switzerland (26) and longer than the average of 4 (IQR: 2–10) days reported in Ethiopia (27). The median time to total delay for PTB patients was 25 (IQR: 13–39) days, which is comparatively shorter than the duration reported in similar surveys and studies conducted both domestically and internationally. For instance, Uzbekistan (28) recorded a median delay of 50 (IQR: 22–92) days, southeast Ethiopia (29) reported a delay of 97 days, while northern Australia (30) observed a delay of 90 (IQR: 60–121) days. The lower reported incidence of PTB in Beijing could be attributed to its higher level of economic development and enhanced accessibility to medical and healthcare services compared to other regions.

As the capital of China, Beijing has one of the lowest TB epidemics in the country and relatively low levels of delay among TB patients. However, our study revealed that 41.7% of patients still experience a delay, and 35.2% of patients encountered a delay in diagnosis. Another study from the Haidian counties of Beijing reported a 54.96% rate of diagnostic delay (31). Our study showed a lower rate of diagnostic delay. This may be related to the timing of our investigation during the COVID-19 pandemic. Screening in health care settings provides an opportunity to find PTB cases and diagnose PTB patients more quickly, as PTB can present with symptoms similar to COVID-19 (32). Additionally, physical examinations such as CT scans are more frequent when screening for COVID-19 in fever clinics, which also aids in detecting PTB cases (33).

The analysis and research findings indicated that passive case finding (PCF), such as transfer, referral, and tracking, are associated with a higher likelihood of delayed diagnosis and medical treatment. These results align with studies conducted in countries with high tuberculosis incidence rates (34, 35). Relevant study has reported that active case finding (ACF) is more effective than PCF in promptly identifying individuals with TB (36). Both the guidelines provided by WHO (37) and China’s tuberculosis prevention and control program (23) emphasize the importance of optimizing the strategy for controlling the source of infection. They suggest added and strengthening new interventions as well as enhancing the screening of high-risk populations and ACF. The investigation findings on factors influencing care-seeking delay and total delay indicate that symptoms such as cough and expectoration lasting for more than 1 week but less than 2 weeks, chest tightness, chest pain, low fever, night sweats, fatigue, loss of appetite, and weight loss were identified as protective factors. These results are consistent with previous studies conducted in Cambodia (38) and Kenya (39). The presence of severe symptoms, such as chest pain, fever, and weight loss, may prompt patients to seek medical treatment (40). Our study also found that being asymptomatic was identified as a risk factor contributing to the delay in seeking medical care. Therefore, it is significant to carry out ACF in high-risk groups for PTB, such as specific populations in high-risk areas of the epidemic. Compared to patients who continued working during their treatment, those who did not need to work were more likely to experience delayed medical treatment and diagnosis. The majority of these individuals were either retired or unemployed, which may have contributed to their reduced inclination to seek medical assistance and limited access to adequate healthcare coverage.

The multivariate analysis results showed that TB specialist hospitals had a lower likelihood of delayed diagnosis. This is because medical personnel and screening technologies in TB specialized hospitals tend to be more vigilant and have a faster ability to identify tuberculosis. In contrast to general medical institutions, they consider whether a patient has tuberculosis after excluding other diseases. Specialized hospitals have higher diagnostic efficiency compared to general health institutions, which often lack the corresponding rapid screening technology. Our study found that the final diagnosis made in tuberculosis control and prevention institutions was a risk factor for diagnosis delay. This may be due to the diagnosis technology of *M.tb* used in these institutions. Certain tuberculosis control and prevention institutions reportedly still rely on conventional laboratory methods such as sputum smear and culture. GeneXpert is a rapid diagnostic method recommended by WHO. Compared to conventional methods for pathogen detection, GeneXpert offers the advantages of rapid detection speed and high accuracy (positive results can be detected when the number of *M.tb* is >130/mL) (41). According to a report, the use of GeneXpert reduced diagnostic delay by 1.79 days (95% CI: 0.27–3.85) and treatment initiation delay by 2.55 days (95% CI: 0.54–4.56) compared to sputum microscopy (42). Currently, GeneXpert technology is only available in certain designated tuberculosis hospitals in China. However, most patients initially seek diagnosis at TB non-specialized hospitals or primary healthcare institutions. Therefore, the implementation of molecular biology detection technology will not only improve the efficiency of identifying PTB patients but also reduce the time required for diagnosis.

Our study has several limitations. Firstly, it was conducted only in two counties of Beijing, which may limit its generalizability to the entire city. Secondly, although we have included most of the data in the system, there are still some variables missing. Additionally, there may be recall bias among certain patients, which could lead to potential differences in their responses to specific questions. These limitations require further comprehensive investigation for future research to address these gaps.

## 5 Conclusion

In summary, PTB patients in Tongzhou and Changping Districts of Beijing are expected to experience relatively low delays in 2021. However, a significant proportion of patients may still face delayed treatment and diagnosis. Therefore, it is crucial to implement effective measures to improve the diagnostic efficiency of tuberculosis, treat patients as early as possible, and reduce patient suffering and economic losses.

## Data availability statement

The original contributions presented in the study are included in the article/supplementary material, further inquiries can be directed to the corresponding authors.

## Ethics statement

The studies involving humans were approved by the Ethics Committee, Beijing Chest Hospital, Capital Medical University, Beijing, China. The studies were conducted in accordance with the local legislation and institutional requirements. The participants provided their written informed consent to participate in this study. Written informed consent was obtained from the individual(s), and minor(s)’ legal guardian/next of kin, for the publication of any potentially identifiable images or data included in this article.

## Author contributions

LZ: Writing – original draft, Visualization, Methodology, Writing – review & editing, Supervision, Project administration, Conceptualization. XM: Writing – review & editing, Writing – original draft, Methodology, Investigation, Formal analysis, Conceptualization. HG: Writing – review & editing, Resources, Data curation. CB: Writing – review & editing, Resources, Data curation. YW: Writing – review & editing, Resources, Data curation. SW: Writing – review & editing, Investigation, Data curation. ML: Writing – review & editing, Investigation, Data curation. YL: Writing – original draft, Writing – review & editing, Visualization, Validation, Supervision, Software, Project administration, Methodology, Funding acquisition, Conceptualization. LL: Writing – review & editing, Visualization, Validation, Supervision, Project administration, Funding acquisition, Conceptualization.

## References

[1] Safdar A. Principles and practice of transplant infectious diseases. New York: Springer (2019). p. 491–502.

[2] World Health Organization. Global tuberculosis report 2018. Geneva: World Health Organization (2018).

[3] Millington KA White RG Lipman M McQuaid CF Hauser J Wooding V. The 2023 UN high-level meeting on tuberculosis: renewing hope, momentum, and commitment to end tuberculosis. Lancet Respir Med. (2024) 12:10–3. doi: 10.1016/S2213-2600(23)00409-5, PMID: 37972624

[4] World Health Organization. Global tuberculosis report 2019. Geneva: World Health Organization (2019).

[5] World Health Organization. Global tuberculosis report 2023. Geneva: World Health Organization (2023).

[6] Peri AM Bernasconi DP Galizzi N Matteelli A Codecasa L Giorgio V. Determinants of patient and health care services delays for tuberculosis diagnosis in Italy: a cross-sectional observational study. BMC Infect Dis. (2018) 18:690. doi: 10.1186/s12879-018-3609-4 30572830 PMC6302482

[7] Teo AKJ Singh SR Prem K Hsu LY Yi S. Delayed diagnosis and treatment of pulmonary tuberculosis in high-burden countries: a systematic review protocol. BMJ Open. (2019) 9:e029807. doi: 10.1136/bmjopen-2019-029807, PMID: 31289094 PMC6629411

[8] Marks GB Nguyen NV Nguyen PTB Nguyen TA Nguyen HB Tran KH. Community-wide screening for tuberculosis in a high-prevalence setting. N Engl J Med. (2019) 381:1347–57. doi: 10.1056/NEJMoa1902129, PMID: 31577876

[9] Disease Control Bureau of the Ministry of Health, Chinese Center for Disease Control and Prevention. Report on the 5th national tuberculosis epidemiological survey in China-2010. Beijing, China: Military Medical Science Press (2011).

[10] Moyo N Tay EL Trauer JM Burke L Jackson J Commons RJ. Tuberculosis notifications in regional Victoria, Australia: implications for public health care in a low incidence setting. PLoS One. (2023) 18:e0282884. doi: 10.1371/journal.pone.0282884, PMID: 36943855 PMC10030020

[11] Supehia S Singh M Bahurupi Y Aggarwal P Chandra R Sharma N. The extent of delay in diagnosis, treatment and their associated factors among tuberculosis patients attending government hospitals of Rishikesh, Uttarakhand: a cross-sectional study. Recent Adv Antiinfect Drug Discov. (2024) 19:137–47. doi: 10.2174/2772434418666230517151828 37198982

[12] Alema HB Hailemariam SA Misgina KH Weldu MG Gebregergis YS Mekonen GK. Health care seeking delay among pulmonary tuberculosis patients in north west zone of Tigrai region, North Ethiopia. BMC Infect Dis. (2019) 19:309. doi: 10.1186/s12879-019-3893-7 30953459 PMC6451246

[13] Getnet F Demissie M Assefa N Mengistie B Worku A. Delay in diagnosis of pulmonary tuberculosis in low-and middle-income settings: systematic review and meta-analysis. BMC Pulm Med. (2017) 17:202. doi: 10.1186/s12890-017-0551-y 29237451 PMC5729407

[14] Makwakwa L Sheu ML Chiang CY Lin SL Chang PW. Patient and heath system delays in the diagnosis and treatment of new and retreatment pulmonary tuberculosis cases in Malawi. BMC Infect Dis. (2014) 14:132. doi: 10.1186/1471-2334-14-132 24606967 PMC3976046

[15] Bojovic O Medenica M Zivkovic D Rakocevic B Trajkovic G Kisic-Tepavcevic D. Factors associated with patient and health system delays in diagnosis and treatment of tuberculosis in Montenegro, 2015-2016. PLoS One. (2018) 13:e0193997. doi: 10.1371/journal.pone.0193997, PMID: 29522545 PMC5844538

[16] Wako WG Wasie A Wayessa Z Fikrie A. Determinants of health system diagnostic delay of pulmonary tuberculosis in Gurage and Siltie zones, South Ethiopia: a cross-sectional study. BMJ Open. (2021) 11:e047986. doi: 10.1136/bmjopen-2020-047986, PMID: 34702728 PMC8549662

[17] World Health Organization. Diagnostic and treatment delay in tuberculosis. (2006). Available at: https://iris.who.int/handle/10665/116501 (Accessed March 15, 2024).

[18] Teo AKJ Singh SR Prem K Hsu LY Yi S. Duration and determinants of delayed tuberculosis diagnosis and treatment in high-burden countries: a mixed-methods systematic review and meta-analysis. Respir Res. (2021) 22:251. doi: 10.1186/s12931-021-01841-6 34556113 PMC8459488

[19] World Health Organization. Global tuberculosis report 2022. Geneva: World Health Organization (2022).

[20] Sun R Wu Z Zhang H Huang J Liu Y Chen M. Assessing heterogeneity of patient and health system delay among TB in a population with internal migrants in China. Front Public Health. (2024) 12:1354515. doi: 10.3389/fpubh.2024.1354515 38371243 PMC10869454

[21] Liu K Ge R Luo D Zheng Y Shen Z Chen B. Delay analysis of pulmonary tuberculosis in the eastern coastal county of China from 2010 to 2021: evidence from two surveillance systems. Front Public Health. (2023) 11:1233637. doi: 10.3389/fpubh.2023.1233637 37637823 PMC10450766

[22] Xia Y Chen H Zhang C Zhao Y Cheng J Zhang H. Guidelines for the prevention and control of tuberculosis in schools: Recommendations from China CDC. China CDC Wkly. (2021) 3:34–8. doi: 10.46234/ccdcw2021.009 34594902 PMC8392894

[23] People’s Republic of China state health and Family Planning Commission. Diagnostic criteria for tuberculosis (WS 288—2017). Electr J Emerg Infect Dis. (2018) 3:59–61. doi: 10.19871/j.cnki.xfcrbzz.2018.01.017

[24] Balasubramnian A Francis PT Leelamoni K Rakesh PS Lalu JS. Diagnostic and treatment delay among new pulmonary tuberculosis patients in Southern India: a cross-sectional study. Indian J Public Health. (2022) 66:S60–s5. doi: 10.4103/ijph.ijph_1079_22, PMID: 36412476

[25] Zão I Ribeiro AI Apolinário D Duarte R. Why does it take so long? The reasons behind tuberculosis treatment delay in Portugal. Pulmonology. (2019) 25:215–22. doi: 10.1016/j.pulmoe.2019.02.005, PMID: 30930122

[26] Auer C Kiefer S Zuske M Schindler C Wyss K Blum J. Health-seeking behaviour and treatment delay in patients with pulmonary tuberculosis in Switzerland: some slip through the net. Swiss Med Wkly. (2018) 148:w14659. doi: 10.4414/smw.2018.14659 30232794

[27] Datiko DG Jerene D Suarez P. Patient and health system delay among TB patients in Ethiopia: Nationwide mixed method cross-sectional study. BMC Public Health. (2020) 20:1126. doi: 10.1186/s12889-020-08967-0 32680489 PMC7368783

[28] Belkina TV Khojiev DS Tillyashaykhov MN Tigay ZN Kudenov MU Tebbens JD. Delay in the diagnosis and treatment of pulmonary tuberculosis in Uzbekistan: a cross-sectional study. BMC Infect Dis. (2014) 14:624. doi: 10.1186/s12879-014-0624-y 25421106 PMC4248454

[29] Hussen A Biadgilign S Tessema F Mohammed S Deribe K Deribew A. Treatment delay among pulmonary tuberculosis patients in pastoralist communities in bale zone, Southeast Ethiopia. BMC Res Notes. (2012) 5:320. doi: 10.1186/1756-0500-5-320 22720757 PMC3434087

[30] Vigneswaran N Parnis R Lowbridge C Townsend D Ralph AP. Factors leading to diagnostic delay in tuberculosis in the tropical north of Australia. Intern Med J. (2023). doi: 10.1111/imj.16223, PMID: 37688576

[31] Wu F Lai C Wang Y Zhang G Li Y Yu S. Tuberculosis infection and epidemiological characteristics in Haidian District, Beijing, 2005-2018. BMC Public Health. (2020) 20:823. doi: 10.1186/s12889-020-08773-8 32487108 PMC7266129

[32] Zumla A Marais BJ McHugh TD Maeurer M Zumla A Kapata N. COVID-19 and tuberculosis-threats and opportunities. Int J Tuberc Lung Dis. (2020) 24:757–60. doi: 10.5588/ijtld.20.0387, PMID: 32912377

[33] Zhang G Yu Y Zhang W Shang J Chen S Pang X. Influence of COVID-19 for delaying the diagnosis and treatment of pulmonary tuberculosis-Tianjin, China. Front Public Health. (2022) 10:937844. doi: 10.3389/fpubh.2022.937844, PMID: 36530737 PMC9755169

[34] Harries AD Lin Y Kumar AMV Satyanarayana S Takarinda KC Dlodlo RA. What can national TB control programmes in low- and middle-income countries do to end tuberculosis by 2030? F1000Res. (2018) 7:1011. doi: 10.12688/f1000research.14821.1, PMID: 30026917 PMC6039935

[35] Mbuthia GW Olungah CO Ondicho TG. Health-seeking pathway and factors leading to delays in tuberculosis diagnosis in West Pokot County, Kenya: a grounded theory study. PLoS One. (2018) 13:e0207995. doi: 10.1371/journal.pone.0207995, PMID: 30485379 PMC6261612

[36] Mhimbira FA Cuevas LE Dacombe R Mkopi A Sinclair D. Interventions to increase tuberculosis case detection at primary healthcare or community-level services. Cochrane Database Syst Rev. (2017) 11:Cd011432. doi: 10.1002/14651858.CD011432.pub2 29182800 PMC5721626

[37] World Health Organization. WHO operational handbook on tuberculosis. Module 2: screening – systematic screening for tuberculosis disease. Geneva: World Health Organization (2021). 33822560

[38] Butsorn A Suggaravetsiri P Tesana N. Delay of treatment among new smear-positive pulmonary tuberculosis patients in Thai-Cambodia border: cases study in Surin and Sisaket Province, Thailand. Res J Med Sci. (2010) 4:340–5. doi: 10.3923/rjmsci.2010.340.345

[39] Kunjok DM Mwangi JG Mambo S Wanyoike S. Assessment of delayed tuberculosis diagnosis preceding diagnostic confirmation among tuberculosis patients attending Isiolo County level four hospital, Kenya. Pan Afr Med J. (2021) 38:51. doi: 10.11604/pamj.2021.38.51.21508, PMID: 33854680 PMC8017359

[40] Sabawoon W Sato H Kobayashi Y. Delay in the treatment of pulmonary tuberculosis: a report from Afghanistan. Environ Health Prev Med. (2012) 17:53–61. doi: 10.1007/s12199-011-0219-9, PMID: 21590428 PMC3258319

[41] Zhang Q Zhang Q Sun BQ Liu C Su AN Wang XH. GeneXpert MTB/RIF for rapid diagnosis and rifampin resistance detection of endobronchial tuberculosis. Respirology. (2018) 23:950–5. doi: 10.1111/resp.13316, PMID: 29691960

[42] Lee JH Garg T Lee J McGrath S Rosman L Schumacher SG. Impact of molecular diagnostic tests on diagnostic and treatment delays in tuberculosis: a systematic review and meta-analysis. BMC Infect Dis. (2022) 22:940. doi: 10.1186/s12879-022-07855-9 36517736 PMC9748908

